# Defensomes, counter-defensomes, and the remodeling of microbial communities

**DOI:** 10.1093/pnasnexus/pgag073

**Published:** 2026-03-17

**Authors:** Vinicius S Kavagutti, Angelina Beavogui, Nicolas Wiart, Patrick Wincker, Pedro H Oliveira

**Affiliations:** Génomique Métabolique, Genoscope, Institut François Jacob, Commissariat à l'Énergie Atomique, CNRS, Université Evry, Université Paris-Saclay, 2 Rue Gaston Crémieux, Evry 91057, France; Génomique Métabolique, Genoscope, Institut François Jacob, Commissariat à l'Énergie Atomique, CNRS, Université Evry, Université Paris-Saclay, 2 Rue Gaston Crémieux, Evry 91057, France; Commissariat à l'Énergie Atomique, Centre National de Recherche en Génomique Humaine, Université Paris-Saclay, 2 Rue Gaston Crémieux, Evry 91057, France; Génomique Métabolique, Genoscope, Institut François Jacob, Commissariat à l'Énergie Atomique, CNRS, Université Evry, Université Paris-Saclay, 2 Rue Gaston Crémieux, Evry 91057, France; Génomique Métabolique, Genoscope, Institut François Jacob, Commissariat à l'Énergie Atomique, CNRS, Université Evry, Université Paris-Saclay, 2 Rue Gaston Crémieux, Evry 91057, France

**Keywords:** mobile genetic elements, horizontal gene transfer, anti-MGE immunity, core defensome, holodefensome, phage therapy

## Abstract

Bacteria and mobile genetic elements (MGEs) have coevolved for billions of years in an enduring evolutionary arms race, leading to the emergence and diversification of a vast arsenal of defense and counter-defense systems. In the last recent years, high-throughput screening methods and genome-resolved metagenomics have markedly enhanced our understanding of the diversity and abundance of immune systems across cultured and uncultured microorganisms. This fueled subsequent interest in better understanding the dynamic tri-kingdom interplay between bacteria, bacteriophages, and eukaryotic cells, and led to renewed efforts to improve alternative antibacterial phage-based therapies. Here, we discuss the evolutionary and ecological dynamics underlying the bacteria–MGE arms race, recent findings on bacterial defensomes, MGE counter-defensomes, holodefensomes, and their key role in the development of microbiome-targeted therapies. To this end, we argue why and how highly conserved anti-MGE defense systems should be prioritized as promising targets for the development of next-generation bacterial inhibitors with broad biomedical relevance, supported by a comprehensive analysis of their distribution and diversity across bacteria.

## Introduction

Horizontal gene transfer (HGT) underpins rapid adaptation to novel ecological niches. This process is primarily driven by mobile genetic elements (MGEs), including bacteriophages (phages), plasmids, and transposable elements, which are pervasive across genomes. MGEs can autonomously transfer between cells via viral particles or conjugation, while encoding beneficial traits to the host (1). For example, prophages often encode important virulence factors in many pathogens, and conjugative elements act as vehicles for the rapid spread of antibiotic resistance genes (2, 3). These adaptive traits enhance the host's fitness in particular environmental contexts, promote evolutionary innovation, and help shape global population structure. However, in other circumstances, MGEs can impose significant metabolic burden on the host, alongside the energetic demands of vertical and horizontal transmission, ultimately reducing its fitness. Consequently, many MGEs are seldom retained in genomes for extended periods, resulting in distinct variations of their repertoires among lineages (4).

The long-term dynamic coexistence of MGEs and bacteria has driven the evolution of sophisticated defense mechanisms to contend and counter these elements. Restriction-modification (R–M) was discovered in the early 1950s (5, 6) as the first anti-MGE immune-like mechanism capable of distinguishing self- from non–self-DNA via the latter's methylation status. It was not until the early 1980s and 2000s that two other widespread anti-MGE defense strategies/systems were respectively uncovered: abortive infection (7) and CRISPR (clustered regularly interspaced short palindromic repeats)/Cas (8). Altogether, they revolutionized the field of genome engineering as precise cleavage/stabilization/editing tools and further propelled the quest for additional defense mechanisms in bacteria as well as MGE counter-defense strategies capable of curbing their action. At the time of writing, approximately 280 defense and counter-defense families have been identified, but the specific mechanisms of action remain in many cases to be fully understood (9, 10).

One field that is primed to take advantage from a thorough understanding of the complex tripartite immune interactions taking place across the phage-bacteria-eukaryote continuum, is that of microbiome editing/remodeling. For example, the application of specifically engineered MGE cocktails (particularly phages) equipped with CRISPR/Cas has provided robust delivery and editing modalities to diverse microbiota both in vitro and in vivo across the biomedical, agricultural, and environmental arenas (11–14). Yet, given the diversity of pathogens in a natural setting and their multiple mechanisms of resistance to CRISPR/Cas (15), such processes remain limited, and would greatly benefit from tailor-made genetic payloads capable of, for example, detouring or inactivating specific host bacterial defense systems with heightened precision and reliability.

In this Perspective article, we discuss recent findings on bacterial defensomes, MGE counter-defensomes, and their interplay with eukaryotic organisms. We also summarize the landscape of defensome conservation across the Bacterial kingdom and specifically point toward defense systems that we consider as promising targets for the development of novel antimicrobials. Finally, we anticipate research avenues and conceptual advances likely to unfold in the coming years.

## Conflicts and alliances in bacteria-MGE-immunity networks

MGEs are pervasive in the genomes of bacteria and believed to have evolved from primordial parasitic replicators since the earliest stages of evolution of life on earth (16). They are semiautonomous symbiotic (commensal, mutualistic, or parasitic) agents of cooperation and conflict, and catalysts of bacterial adaptation and evolutionary diversification. Apart from carrying traits essential for their replication, MGEs may also convey adaptive genes capable of enhancing host fitness or niche adaptation (e.g. antibiotic resistance, virulence factors, secondary metabolites). Despite the potentially positive effects in acquiring such public goods, carrying a selfish MGE with misaligned evolutionary interests can incur a hefty and sometimes deadly cost to the host cell. For example, virulent phages might kill the cell to release their progeny, while the acquisition of other elements such as plasmids, integrative conjugative elements (ICEs)/integrative mobilizable elements (IMEs) or transposable elements can decrease the host's growth rate or disrupt chromosomal organization (17).

Conflicts and alliances also take place between MGE families, and their fitness effects can be as significant as those stemming from host-MGE interactions. One notable example of hyperparasitism (a parasite of a parasite) is that of phage satellites, small mobile elements unable to produce virions, that hijack the capsid of functional helper phages (18). Two recent studies uncovered a plethora of very diverse phage satellites (∼5,000) in complete bacterial genomes (19) and a non-negligent proportion (∼0.6%) of marine viral particles as likely encapsidated satellites (20). Another example refers to MGEs that do not encode a functional conjugative machinery and as consequence require those encoded by conjugative plasmids and ICEs. The former includes mobilizable plasmids encoding a relaxase and an *oriT*, plasmids carrying only an *oriT*, and IMEs (21). In some cases, hyperparasitism may impact the fitness of MGEs. If the impact on a parasitized MGE is detrimental to bacteria, then the hyperparasite may end up benefiting the host (or vice versa).

Occasionally, the frontier between MGE families gets fuzzy, as some categories appear to contain features belonging to multiple genetic elements without a strict delineation of identity or function. For example, hybrid elements were found to result from recombination between phages with other MGEs, such as plasmids, transposons, and genomic islands (22). The most well studied are phage-plasmids, an evolutionary diverse and likely ancient MGE category accounting for ∼5% to 7% of all phages and plasmids, and capable of transferring horizontally between cells as phages and vertically within cellular lineages as plasmids (23, 24). The unique biology and fluid identity of hybrid MGEs raises important questions concerning: (i) the trade-offs or fitness costs associated with lifestyle diversification and (ii) their breadth of interactions with the host and an increasingly crowded network of MGEs.

A third aspect to be considered when dealing with networks of conflict-alliance in bacteria, is the tight evolutionary entanglement between MGEs and immune systems, seemingly rooted on complementary properties of selfishness and mobility. In bacterial genomes, defense systems and MGEs tend to colocalize in defense islands, which favor genetic exchange and functional versatility of their corresponding machineries. Evidence for such intertwining can be observed, for example, in the exaptation of transposases and integrases encoded by MGEs for defensive functions, or in the recruitment or repurposing of defensive components (e.g. CRISPR/Cas, nucleases, MTases) by MGEs either for their stabilization or within the frame of inter-MGE conflicts (25). Strikingly, the anti-MGE activity of phage satellites can confer a selective advantage to their helper phages during competition with virulent phages, effectively converting an otherwise parasitic interaction into a mutualistic one (26). Such directional interplay between MGEs and defense systems and concomitant exchange of genetic components effectively reconciles the paradox of genetic parasites' emergence and inevitability, despite their high cost to the host and evolution under negative selection. In this regard, it remains to be determined the role of arms race de-escalation, in particular how regular loss of immunity can sustain a viable MGE population. It also remains to be clarified the extent to which mobility of defense genes benefit the gene itself, the associated MGE, the donor, or the recipient host, and how such trends effectively change with important confounding factors such as ecological context and defensome family (27–29).

## Defensomes and counter-defensomes

**Figure 1:**
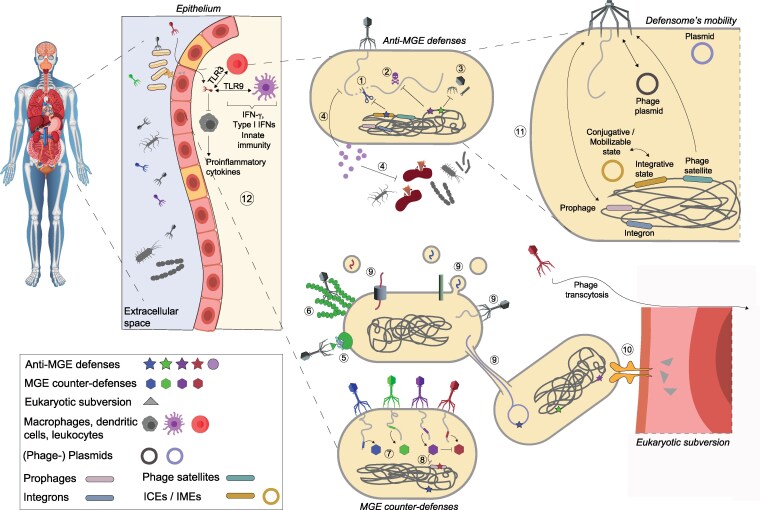
Defensomes, counter-defensomes, and interplay with a eukaryotic host's immune system. 1: R–M systems discriminate self from non–self-DNA through methylation. 2: abortive infection (Abi) in which the infected cell undergoes programmed cell death before the invading phage can complete its replication cycle. 3: Inhibition of virion assembly (e.g. Tail Assembly Blocker, Tab). 4: Inhibition of bacterial replication. These include, for example, anti-MGE and antibacterial small molecules, anthracyclines, aminoglycosides, or chain terminators produced by prokaryotic viperins. Aminoglycosides have both antibacterial and antiphage properties. 5: Prevention of phage adsorption due to receptor post-translational modifications (glycosylation) or receptor modification through interaction with other proteins. 6: Prevention of phage adsorption due to receptor occlusion by surface structures (glycan capsule). 7: MGE counter-defense (e.g. anti-nucleic acid degradation, anti-Abi). 8: Counter-defense–associated genes can trigger Abi loci encoded by MGEs (e.g. prophages). 9: Conjugation, transfection, transformation. 10: Eukaryotic subversion, for example undertaken by nucleomodulins. 11: Defense systems can be carried by a multitude of MGEs. The latter include (i) integrative conjugative elements, which can be excised under specific environmental conditions and transferred via conjugation; (ii) lysogenic phages, capable of integrating into the host chromosome; (iii) phage satellites, which lack structural components of virions and rely on helper phages for their own transmission; (iv) plasmids; (v) phage-plasmids; and (vi) integrons. 12: Phages function as ligands for pattern recognition receptors such as Toll-like receptors (TLRs). Viral nucleic acids can be detected by TLR9, which senses DNA; TLR7/8, which detect single-stranded RNA; and TLR3, which recognizes double-stranded RNA. These nucleic acid–sensing TLRs can trigger, among other responses, the production of type I interferon (IFN). Phages can be internalized by leukocytes leading to the production of type I IFN, or uptake by dendritic cells leading to the production of IFN-γ. Some phages are known to induce production of proinflammatory cytokines and activate phagocytic cells, such as neutrophils and macrophages. Image credits (copyright-free): anatomical structure and bacteriophage (Matt Cole/Vecteezy), bacteria (macrovector_official/Freepik), macrophage and dendritic cell (Md Mijanur Rahman/Vecteezy), leukocyte (Fitri Handayani/Vecteezy).

It can be argued with some authority that the inevitability and evolutionary emergence of defense systems and cell immunity (innate, adaptive, and programmed death) are a common thread across all life forms that most likely arose at the earliest stages of evolution. It seems plausible that among the first primordial gene pools, coevolution between primitive replicator systems and parasitic cheaters fueled specific forms of compartmentalization followed by the emergence of more complex multicomponent defense systems, pattern recognition sensors, and ultimately, complex adaptive immune systems (25). Greatly propelled by the successful repurposing of bacterial immune systems as genetic engineering tools, the last decade has witnessed the identification and, in some cases, the mechanistic characterization of an extensive arsenal of previously unknown anti-MGE defense systems [reviewed in (30, 31)]. These systems can be deployed at various stages of the MGE infection process, by degrading invading nucleic acids, inhibiting their replication, or inducing dormancy or death of infected cells to stop the mobile element's spread through the microbial population (Fig. 1). Certain defense systems can target multiple families of MGEs, while others seem more specialized. For example, Ddm systems can work together or independently to defend a population against both plasmids and bacteriophages. Type II Lamassu systems DdmABC offer protection against bacteriophages and large low-copy-number conjugative plasmids through a dual-function abortive infection mechanism, while DdmDE rapidly degrades smaller, multicopy plasmids, regardless of their origin of replication (32–34). Other immune pathways stand in stark contrast to those described previously, either at the mechanistic level or by means of the chemical nature of the molecules involved. For example, defense-associated reverse transcriptase systems seem to disrupt conventional notions about the defining features of protein-coding genes and subvert the conventional flow of genetic information in a cell. Defense-associated reverse transcriptases operate an ingenious mechanism based on the rolling circle reverse transcription of noncoding RNA templates, which become double-stranded upon viral infection, and lead to de novo synthesis of nearly endless open reading frame (*neo*) genes whose expression halts cell growth and restrict viral spread (35, 36). Another example concerns the recent findings on the yet poorly explored anti-MGE chemical defensome. These include DNA intercalating agents such as the anticancer drugs daunorubicin, doxorubicin, epirubicin, and idarubicin who block phage replication (37), as well as lanthipeptides (branded lantiphages) found in Actinobacterial defense islands and capable of modulating the viral transcriptional program (38).

Many novel defense systems have been uncovered through bioinformatic exploration of reference genome databases (e.g. National Center for Biotechnology Information [NCBI] Reference Sequence Database) (9), but the latter overrepresent organisms amenable to laboratory cultivation and, therefore, provide a limited snapshot of the fraction of uncultured environmental microbial diversity. A recent large-scale screening of high-quality bacterial population genomes reconstructed from environmental metagenomes highlighted the diversity of defensomes and the potential for functional cooperation and generation of novel functions between different defensive modules (27). In another study, the use of eDNA libraries as a strategy of unearthing novel antiviral systems allowed to isolate a DNA glycosylase from an unknown organism that provides immunity through the excision of α-glucosyl-hydroxymethylcytosine nucleobases present in the T4 genome, thus inhibiting phage replication after infection (39). Additional mechanisms of defense are expected to be found by targeting a larger breadth of microbial communities across multiple biomes.

As the number of anti-MGE defense families identified in bacteria expanded, so has the discovery of MGE-encoded systems capable of counteracting them. Such counter-defensome deploys multiple mechanisms to inactivate host immune systems (beyond bacteriophage gene mutations), that include: (i) direct binding to immune proteins [e.g. anti-R–M (40), anti-CRISPR (Acr) (41, 42), anti-Gabija (43), anti-BREX (44), anti-RecBCD (45), anti-T–A (46), anti-SIR2 (47)]; (ii) post-translational modification of immune proteins (e.g. anti-T–A, Acr); (iii) targeting of secondary messengers [e.g. anti-CBASS (48), anti-Pycsar (48), Acr, anti-Thoeris (49), anti-Retron (50)]; and (iv) counteracting metabolite-depleting defense systems (e.g. NARP1-2) (51). Other counter-defense systems such as anti-AVAST (47) and anti-Hachiman (49) operate through mechanisms that remain unknown or poorly characterized. This repertoire of MGE counter-defensome has been recently reviewed and expanded (10, 52, 53), and as of today it encompasses >150 distinct systems. The diverse repertoire of counter-defensomes seems capable of constraining or specifically fine-tuning the MGEs' host range to bacteria harboring varied defensomes (46). The former are often deployed to serve the MGE’s own interests (which frequently transcend those of their hosts), including the establishment of the MGE during its early stages of acquisition in the recipient cell (53) or in mediating inter-MGE warfare activities (54–58). Interestingly, MGEs also seem to benefit from promoting the spread of defense systems as a preemptive strategy against competing MGEs with their own counter-defense. For example, phages have been described to mobilize defense mechanisms encoded by phage satellites (59), plasmids (60), or chromosomal islands (61). Similarly to defensomes, there is mounting evidence that MGEs cluster their counter-defensome in islands. For example, Acr were reported to colocalize with antagonists of other defense systems such as anti-R–Ms (62). In plasmids, diverse counter-defense islands were found to be enriched in the leading strand's “stability” region adjacent to propagation genes and the *oriT*, presumably because they must be rapidly expressed in the earliest stages of conjugation (53).

Concomitant to the unraveling of bacterial immune defense and MGE counter-defense families was the realization of their conservation across domains of life. Some examples of this conserved ancestral immunity, include proteins implicated in pathogen detection (cGAS/STING), activity against transposons (PLD6/MOV10L1), signal transduction (TIR domains), antiviral effectors (GIMAPs, FHAD1/EFHD2/CTRC, viperins), and viral immune evasion proteins such as cGAMP PDE/Acb1 (63–65). It is foreseeable that the next years will see broader initiatives to expand the known repertoire of defense and counter-defense, a better understanding of how such mechanisms collaborate or antagonize with one another, and the concomitant extension of the set of shared rules that govern host-MGE interactions across multiple branches of the tree of life (10, 66).

## The holodefensome: Clinical and environmental relevance

With the increase in whole-genome sequencing initiatives and access to long-read technologies, the detection of HGT events can be better captured, reducing misincorporation of contaminant DNA sequences in assemblies derived from short-read data. Initially controversial and highly debated, signatures of HGT in eukaryotic genomes (e.g. insects, plants, fish, fungi) have now been documented and substantiated by multiple independent studies, demonstrating its enduring influence in evolution across all branches of the tree of life (67). One example is bacterial-to-eukaryotic HGT, potentially mediated by vesicles or nanotubes, which may facilitate the propagation of immunogenic cargos and ultimately endow the host with novel functional and ecological roles as well as capacity to adapt to specialized niches (68, 69). Additional routes for genetic exchanges capable of expanding the genetic pool accessible to eukaryotes rely on bacteriophages' ability to be internalized into the former's genome. For example, infected bacteria containing phage DNA harboring 5′-linked terminal proteins are capable of resisting exonucleolytic degradation and, by means of the terminal protein's nuclear localization signals, be transported inside the host cell nucleus (70). In another study, the *Escherichia coli* phage PK1A2 was able to recognize and bind neuroblastoma cells presenting polysialic acids on their cell surface. Following adhesion, the phage was internalized by the endolysosomal pathway and ultimately degraded (71). It is posited that during such internalization bacteria and phages may evade lysosomal degradation, fueling opportunities for trans-kingdom genetic exchange and stimulation of cellular immunity (72–75).

The previous findings add to an increasing appreciation of eukaryotes as composite entities—holobionts—in which commensal, symbiotic, and pathogenic microorganisms share a common host. The discriminatory sorting of species orchestrated by the ensemble of immune systems present in these metaorganisms, shapes ecological relationships, and helps delineating intermicrobial dynamics. At this point, it seems appropriate to introduce the concept of holodefensome as the complete set of defense (immune) systems, and their multidirectional and nonlinear crosstalk across microbiome, virome/phageome, and eukaryotic hosts. In fact, the rational modulation/redesign of the specificity and magnitude of the holodefensome crosstalk is currently one of the major bottlenecks to the successful engineering of complex microbial consortia across clinical and environmental settings. Let's take phage therapy as example: its likelihood of success greatly depends on the extent of immune evasion (or compatibility) achieved between the complex, tripartite components of the holodefensome arsenal (76–78). Such immune interplay can unfold at multiple levels. First, the immune system of the plant or animal host can directly recognize invading phages, eliciting an increase in the production of chemokines or antiviral cytokines like interferon γ and interleukin-12 (79, 80). In this sense, eukaryotic association modules encoding protein domains and cleavage sites key to eukaryotic functions have been reported in bacteriophage genomes (81), suggesting events of lateral gene transfer across these branches of the tree of life. Also, phage ankyrin proteins were shown to undermine eukaryote immune responses against bacteria and facilitate bacteria-eukaryote coexistence through reduced phagocytosis rates (82). Second, phage-mediated lysis of bacteria can trigger an immune response to the contents of the bacterial cells (e.g. lipopolysaccharides). Third, phages may also switch between lysogenic/lytic states or modulate its activity and with this alter the composition, functionality, adaptability, and fitness of the resident microbiome (83). In this regard, evidence is slowly emerging that a Goldilocks effect might be at play in which moderate rates of phage-induced lysis sustain a microbiome structure that is most resistant to pathogen invasion (84). Finally, the growing repertoire of anti-MGE defensome strategies utilized by bacteria call for innovative and translational insights to develop effective therapeutics. Previous work provided proof of concept on how to target antibiotic resistance-conferring replicons by means of specifically designed engineered phages or plasmids. However, these attempts resulted in less than perfect delivery/conjugation efficiencies, especially in complex microbial communities like the human gut (83, 85–87). While extremely insightful, such strategies do not account for potential vulnerabilities of the designed MGEs to the host defensome. Conceivably, the tailored design of counter-defense payloads curbing the defensome mechanisms found within certain bacteria of interest can ultimately improve conjugation efficiency in nonviral biopharmaceuticals or result in more effective phages with precise host ranges that will be more tailored for phage therapy. As a case in point, phages artificially incorporating inactivators of Druantia type I effectively and specifically eradicated bacteria harboring this system (47). The continuous development of artificial intelligence and machine learning suggests that its predictive power will become central in developing custom-built models for predictive phage therapy, design of personalized phage cocktails for patients, and delivery of best combination of counter-defensome cargo in synergy with the host immune system (88, 89). Analogously to MTases with a well-characterized involvement in critical functional roles of bacteria, it appears likely that the targeted inhibition of other defensome modules will open additional directions enabling precision microbiome targeting. In the next section, we draw on a comparative genomics analysis and shortlist a set of candidate defensome gene families to be prioritized for further studies.

## Remodeling of microbiome composition via core defensome targeting

**Figure 2:**
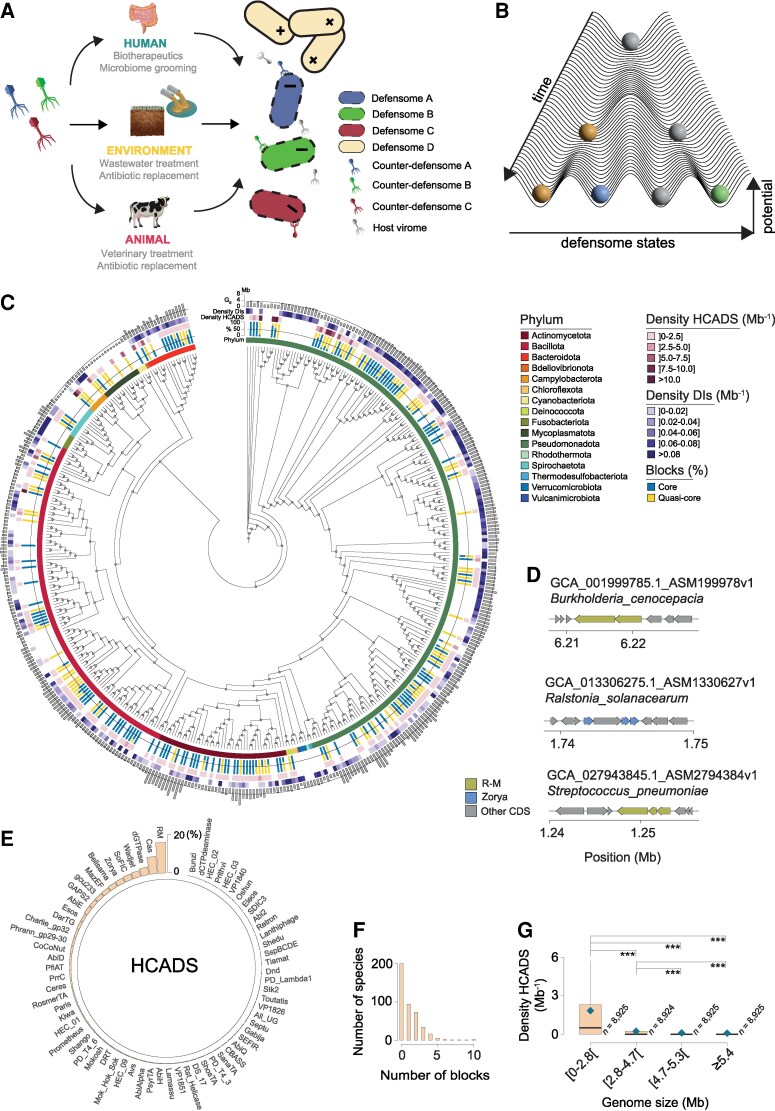
HCADS in bacteria. (A) Potential applications of phage therapy cocktails from the One Health perspective. Such cocktails can be designed to harbor counter-defensomes specifically targeting bacteria with compatible defensomes (e.g. anti-R–M versus R–M). (B) Adaptation of the Waddington's epigenetic landscape concept to defensome evolution. The z-axis represents the hills of potential energy separating different defense systems, which are a measure of the promiscuity/redundancy in defensome composition. The 2-dimensional plane spanned by the x and y axes represents the defensome state/complexity space. As lineages diverge over time, so does their defensome composition (colored spheres correspond to different families of defense systems). Progression through the landscape is directionally unrestrained, which is key to produce the defensome diversity required for bacterial anti-MGE protection. Natural selection will tailor the level of defensome variation to the needs and challenges posed by the lifestyle of the species. In some cases (e.g. gray spheres), defense systems are retained over time across all members of a given taxonomic rank (HCADS). (C) Phylogenetic cladogram representation of 429 bacterial species, their corresponding phyla, normalized % of HCADS blocks (core [100%] in blue, quasi-core [90%–100%] in yellow), density (per Mb) of HCADS, and density (per Mb) of defense islands (DIs). The distribution of average genome sizes (Mb) is shown as outer layer barplots. (D) Representative examples of HCADS in *B. cenocepacia* (type III R–M), *R. solanacearum* (type III Zorya), and *S. pneumoniae* (type I R–M). (E) Percentage of bacterial genomes harboring each HCADS family. (F) Distribution of the number of HCADS blocks (per species) across our dataset. (G) Variation in density (per genome and per Mb) of HCADS with genome size (Mb) for our dataset. Null values were included. Boxplots show the 25th to 75th percentiles, with the median indicated by the central black line. Whiskers extend to 1.5× the interquartile range, and individual data points were removed to improve visualization. Statistical significance was assessed using a 2-sided Mann-Whitney-Wilcoxon test. *P* values are indicated as ***P < 10^−3^. The number of genomes (analyzed are shown next to each boxplot. Image credits (copyright-free) for panel A: bacteriophage (Matt Cole/Vecteezy), intestines (brgfx/Freepik), wastewater (macrovector/Freepik), soil (brgfx/Freepik), cow (pikisuperstar/Freepik). CDS, coding sequence.

In light of the growing threat posed by drug-resistant bacteria, a rekindled interest in phage-based antibacterial therapy is gaining momentum as a suitable and systemic therapeutic avenue (90). Such scenario provides ample opportunities to potentially reshape microbiome composition using microbe-specific phages under the frame of the intersectoral One Health concept. These opportunities include, for example, the treatment of bacterial infections, surface disinfection, biofilm control, wastewater treatment, and their use as substitute or adjunct to antibiotics in veterinary medicine, animal husbandry, agriculture, and aquaculture (Fig. 2A) (91). The previous begs the question of which bacterial genes should be prioritized for the development of antimicrobials capable of targeting, for example, an entire species? One possibility relies on persistent genes, defined as orthologs shared by all (or nearly all) members of an evolutionarily coherent group, likely encoding functions under purifying selection. Such genes (many of them essential) are potential targets to develop broad-spectrum antibiotics or vaccines that could target an entire bacterial species (Fig. 2B). Bacterial anti-MGE defensomes were recently characterized in species from reference genome databases (e.g. NCBI Reference Sequence Database) (9) and in complex communities from soil, marine, and human gut environments (27). While the elucidation of the mechanistic principles underpinning the defensome remains an area of active research, it is now known that some of these systems present an evolutionary dynamics and a broader set of cellular functions unrelated to immunity. Also, our current understandings do not disentangle between the core and accessory layers of the defensome, and the few efforts to delve into this question remained limited to single bacterial species or precise anti-MGE families (92, 93).

Motivated by these considerations and by the prospect of shortlisting core defensome families as targets for microbiome remodeling, we conducted an in-depth investigation on its abundance, distribution, and diversity in bacteria (**Methods**). We started by defining highly conserved anti-MGE defense systems (HCADS) as persistent (core + quasi-core) complete defensome modules present in at least 90% of the genomes of a species (Fig. S1). We found 46,463 HCADS in 35,699 bacterial genomes from 429 species (considering only those with at least 10 complete genomes available in GenBank) (Fig. 2C; Tables S1–S4). This represents 13.6% of all complete defense systems found. Core HCADS were almost ubiquitous across bacterial large phyla, and more abundant than quasi-core ones (57 versus 43%) (Fig. 2C; Table S5). Some notorious examples of species harboring HCADS include the human pathogens *Burkholderia cenocepacia* and *Streptococcus pneumoniae* or the plant pathogen *Ralstonia solanacearum* (Fig. 2D). Some of the most predominant families of HCADS (e.g. R–M, CRISPR-Cas, dGTPases) (Fig. 2E, Fig. S2A; Table S6) are also among the most pervasive across bacteria (9, 27). Most species analyzed (52%) are devoid of HCADS, but some like *Moraxella bovis* or *Streptomyces clavuligerus* appear as outliers with as much as 10 HCADS blocks per genome (Fig. 2F). We observed negative correlations between the density of HCADS and genome size (Fig. 2G). We can speculate that bacteria with more streamlined genomes (e.g. several endosymbionts or intracellular parasites) typically engage in less HGT and have higher proportion of core genes, among which defense-related ones got retained and assumed key roles over time. On the contrary, species with larger genomes typically show a considerable turnover of defense genes, which tend to be kept in the accessory genome to reduce metabolic burden. Concomitantly, species with lower average nucleotide identities (ANI), indicative of greater evolutionary divergence, tend to harbor fewer HCADS blocks (Fig. S2B). In contrast, HCADS abundance does not vary with the number of genomes in species, consistent with a limited sampling bias (Fig. S2C).

Despite defense systems preferentially localizing in the accessory genome, we nevertheless tested the colocalization of HCADS in defense islands, as well as their association with different mechanisms of genetic mobility (**Methods**). We observed 1.2% of HCADS colocalized in defense islands (Table S7), despite the latter's known role as high-turnover sinks of genetic diversity. Their defensive content was very diverse (Fig. S3A), with some families of HCADS (e.g. Gabija, SDIC3, Septu) being overrepresented compared with regions outside defense islands (Fig. S3B). As it would be anticipated, HCADS are predominantly chromosomally located (excluding MGEs) (Fig. S3C; Tables S8,S9). The small amount (3.3%) carried by MGEs shows a slight but significant preference for colocalization with ICEs/IMEs/plasmids compared with integrons and prophages, suggesting a role other than host defense (e.g. maintenance, transmission, or inter-MGE conflicts).

In a follow-up of recent observations of sedentary chromosomal integrons as biobanks of bacterial defense systems (94), we further observed higher HCADS densities in the former compared with mobile integrons (Fig. S3D). When investigated at a more granular level, we observed a highly heterogeneous landscape of combinations of families of HCADS/MGE class (Fig. S3E), in what can presumably be linked to different degrees of MGE specialization and to a dynamic and multilayered interplay with shifting allegiances. For example, Kiwa and Shango were respectively (and uniquely) overrepresented in ICEs and prophages, whereas other defensome families such as AbiD, R–M, and dGTPases were overrepresented across multiple classes of MGEs. Alongside to these findings, MGEs also seem to have retained highly conserved counter-defense systems (HCCDS). As an illustrative example, upon screening of the entire human Gut Phage Database (95), we found 10 viral operational taxonomic units (vOTUs) (with at least 10 genomes each) having anti-R–M, anti-CRISPR, and anti-Thoeris as HCCDS (see **Methods** and Fig. S3F; Table S10). Such HCCDS are expected to counteract the highly abundant R–M, CRISPR, and Thoeris defensome families already found in human gut bacteria (27).

Based on the previous information and particularly the bacterial HCADS list, it would appear logical to attempt to suppress or modulate their activity. Given that most HCADS identified rely on DNA methylation, a potential avenue for rational microbiome editing would be the epigenetic targeting of specific bacterial subpopulations through inhibition of the catalytic domains of SAM (AdoMet)-dependent DNA MTases (Fig. S3G). Depending on system architecture, such targeting would be expected to induce REase-mediated degradation of the bacterial chromosome in type II systems, or to impose a substantial fitness cost (manifested as loss of viability, virulence, or stress tolerance) in type I, III and solitary MTases, as previously demonstrated (96–98). High-throughput screening of SAM analogs across a broad spectrum of HCADS could therefore enable the development of potent and selective inhibitors, and open new possibilities for the development of species-specific epigenome-targeting drugs (99).

Hence, bacteria possess a diverse repertoire of HCADS, the patterns of their distribution are very diverse and dependent of genome size, taxonomy, and colocalization with MGEs and defense islands. Targeting the catalytic site of the widespread HCADS' SAM-dependent MTases thus appears as a first step to achieve species-level remodeling of complex microbial communities. At a broader level, establishing the foundation for scalable HCADS/HCCDS-based therapies aimed at recalibrating microbial ecosystems will require sustained discovery of previously uncharacterized systems (e.g. much of the counter-defensome remains unknown), as well as a rigorous understanding of their functional specificity through informed design (e.g. anti-R–M are not universally effective across all R–M architectures).

## Conclusion

Defense systems are known to make up a substantial fraction of the bacterial accessory genome, reflecting the fitness costs with anti-MGE resistance under dynamic predation pressures. However, some defense genes and systems seem much more conserved, either denoting a specialization in protection or a broader role across multiple cellular functions. A few known examples include the RecBCD exonuclease involved in the repair of double-strand breaks by homologous recombination, several type II solitary MTases, among others (93). Such cases might have arisen to provide evolutionary advantage under a specific environment or genetic landscape. Presumably, a subset of recently acquired genes gradually dominated and assumed core essential functions either via MGE domestication (100), or by hijacking critical genome-encoded features of metabolism and virulence (101). Alternatively, epistatic interactions with genes carried in MGEs might have triggered the essentiality of already existing core genes (102). Based on the idea that the presence of these core genes in most of the genomes of a clade indicates that they are likely to participate in key bacterial processes, it seems logical to assume that they would serve as ideal targets for the identification of inhibitors with antibacterial activity. Here we showed that multiple defensome families, particularly abundant ones such as R–M, are HCADS in bacteria. Some notorious bacterial species harboring such HCADS include the human pathogens *B. cenocepacia*, *Neisseria meningitidis*, and *S. pneumoniae* (Fig. 2D).

In therapeutic contexts, phage-bacterium dynamics are deeply intertwined with, and influenced by, concurrent selective pressures exerted by the eukaryotic host immune system. The latter's competence not only governs vulnerability to bacterial pathogenesis, but also dictates the therapeutic outcomes of phage-based interventions. These multifaceted interactions form a tripartite ecological and evolutionary system, the dynamics of which are orchestrated by a layered, context-dependent crosstalk: (i) phages and bacteria engage in a tug of war of defense and cycles of (counter)_n_ defense; (ii) shifts in phage-bacteria population structure potentiate host innate immune activation through direct interfacing with the cell membrane receptors, thereby augmenting phagocytosis and eliciting proinflammatory cytokine and chemokine responses; and (iii) the striking evolutionary conservation of immune modules across prokaryotes and eukaryotes reveals potential for cross-kingdom immunomodulation, wherein viral-encoded inhibitors of bacterial defenses may suppress analogous eukaryotic pathways while simultaneously opening avenues for engineering potentiator mutations in antiviral factors and for exploiting prokaryotic effectors to antagonize eukaryotic viruses.

Developing antimicrobial or microbiome remodeling drugs poses great challenges, as evolutionary pressures inevitably erode treatment efficacy. Resistance must therefore be anticipated for repeated or longer-term use of defense/counter-defense modules or phages in therapy because it may be readily acquired and rapidly disseminated through bacterial populations just as MGEs have led to an unanticipated rise in antibiotic resistance. For example, intrinsic and acquired resistance was frequently observed as an immediate response to in vitro treatment of bacteria with episomally encoded CRISPR/Cas9-based antimicrobials (103). Other studies caution for the uncontrolled generation of bacterial populations capable of resisting multiple phages and advocate for complementary strategies for mitigating bacterial resistance, including the use of highly efficient phages, cocktails, phage training, and engineering (104–106). Moreover, targeting a focal pathogen with phages can have unintended ecological consequences when the pathogen competes with other species not targeted by the phage. In such cases, phage therapy (or other “narrow spectrum” approaches) may result in competitive release of previously rare pathogens. This implies that “narrow spectrum” antimicrobials, such as phages, may not always be the most effective option when multiple pathogen species coexist within a polymicrobial infection. We can identify several outstanding questions that demand thorough investigation in the forthcoming years. These include: (i) a better understanding of how anti-MGE defense genes horizontally transferred from bacteria to primordial eukaryotes, and whether such rare events may have been a substantial driver of multicellularity; (ii) improving the overall prediction accuracy of defensomes and counter-defensomes, and their integration into unified frameworks for virus–host prediction; (iii) deeper knowledge on how defense or counter-defense operates in a redundant, antagonistic or synergistic fashion, their cost/benefit ratio across different environmental niches, and their function on spatial community structure; (iv) novel multidisciplinary research efforts to advance our understanding on the holodefensome tripartite interplay between phages, bacteria, and the mammalian immune system, particularly in the clinical and environmental arenas; and (v) quantitatively determining the efficiency of defensome-based editing in complex microbial consortia, and identifying the factors that limit and promote the penetrance and resilience of such editing. Our list of HCADS provides a first stepping stone in such a direction.

## Methods

### Data

We downloaded all latest, full, and complete bacterial genomes available at NCBI GenBank (last accessed in March 2025). We first assessed the quality of genomes using CheckM v1.2.3 (options: taxonomy_wf domain Bacteria -x fna) and retained only genomes with a completeness ≥90% and contamination ≤5%. To reduce conspecific redundancy, species were de-replicated at an ANI threshold < 100% using dRep (107) v3.5.0 (options: -pa 1). We further proceeded our analyses with species having at least 10 genomes available. The final dataset encompassed 35,699 genomes pertaining to 429 species (Table S1). Sequence annotation was performed using Prokka (108) v1.14.5 with default parameters.

### Identification of anti-MGE defense systems and islands

Anti-MGE defense systems' detection was performed with DefenseFinder (9) v2.0.0 using models from v2.0.2 (6d08497, February 7, 2025) along with default parameters (only complete systems were used in our study). Species for which all genomes yielded no hits against DefenseFinder were excluded for core defensome analysis. Defense islands were defined as clusters of defense systems separated by no more than 10 genes and containing at least 5 genes representing a minimum of 3 distinct defense families.

### Identification of highly conserved systems

For each bacterial species, protein sequences corresponding to the full spectrum of complete anti-MGE systems identified by DefenseFinder were selected for comparison in an all-vs-all manner using BLAST+ (109) v2.12.0 (default parameters). Two sequences were acknowledged orthologs if a reciprocal best hit existed among them, and both hits had percentage identity ≥ 95%, ≥80% coverage, and e values ≤10^−4^. To identify HCADS and blocks, we computed core defense systems (100% presence) and quasi-core systems (90%–99% presence) across all genomes of each species (Table S1). In this study, we considered presence/absence of HCADS irrespectively of genomic position, meaning that blocks may contain syntenic and nonsyntenic HCADS. To identify HCCDS, we downloaded the complete set of viral contigs from the Gut Phage Database (95), totaling 142,809 contigs. ANI values were calculated with the anicalc.py script from the CheckV repository (110). Viral contigs were then clustered into viral operational taxonomic units (vOTUs) using aniclust.py (options: –min_ani 95 –min_tcov 85 –min_qcov 0), resulting in a total of 992 vOTUs. Gene prediction and functional annotation were performed using Prokka with default parameters, followed by DefenseFinder (option: –antidefensefinder-only to identify counter-defense systems). HCCDS were predicted from the annotated datasets following the same methodology described previously for HCADS.

### Identification of mobile genetic elements

Plasmids were detected through the use of PlasClass (111) v0.1.1 under default configurations. Only positive hits with a score equal to or greater than 0.8 were considered. Integrons were detected using IntegronFinder (112) v2.0.2 (−local_max). Prophages were detected with Virsorter2 (113) v2.2.4 (−include-groups dsDNAphage,ssDNA –min-length 5000 –min-score 0.9). ICEs and IMEs were identified using the default settings of ICEfinder (114) v1.0. To avoid confounding effects in the defensome-MGE colocalization analyses, any instances of overlapping MGEs (e.g. integrons carried by plasmids) were excluded. Such cases represented less than 8% of the total MGE dataset.

### Phylogenetic cladogram and average nucleotide identity

We scanned the full collection of 430 genomes (429 + outgroup) for the presence of 120 conserved protein HMM markers (115) using hmmsearch. Alignment of amino acid sequences for each marker was performed with MAFFT (116) v7.490 and trimmed using BMGE (117) v2.0 (parameters: -t NT -g 0.5 -b 3 -m ID). Utilizing IQ-TREE2 v1.6.12, we constructed a maximum likelihood phylogenetic cladogram employing parameters (-st DNA -bb 1000 -alrt 1000 -m TEST) and conducting 1,000 iterations of ultrafast bootstrapping (118) and SH testing (119). For model selection, the ModelFinder (120) option of IQTREE was activated, and *Haloquadratum walsbyi* DSM 16790 (GCA_000009185.1) was chosen as the outgroup. Species-level divergence was measured by computing ANI across all genome pairs using FastANI (121) v1.34.

### Structural modeling

Prediction of HCADS' MTases 3D structure was performed with AlphaFold3 (122) on the AlphaFold server using default seed autogeneration. Resulting crystallographic information files (.cif) were used as input to ChimeraX (123), and corresponding pdb files were exported.

### Statistical and graphical analyses of data

All statistical and graphical analyses were conducted using R v4.3.1 (R Foundation for Statistical Computing).

## Supplementary Material

pgag073_Supplementary_Data

## Data Availability

All data supporting the findings of this study are available within the article and its supplementary files. A dedicated docker image named BARCODE (BActeRial COre DEfensome) allowing to reproduce all key analyses of this work is publicly available at https://github.com/oliveira-lab/BARCODE.
