# Supplementary material for: Defensomes, counter-defensomes, and the remodeling of microbial communities
Source: PNAS Nexus. 2026 Mar 17;5(4):pgag073. doi: 10.1093/pnasnexus/pgag073 (PMC13064858; doi:10.1093/pnasnexus/pgag073)
Supplement: pgag073_Supplementary_Data [file pgag073_supplementary_data.zip › PNASNEXUS-PNASNEXUS-2025-01355R-s02.pdf]

Defensomes, counter-defensomes, and the remodeling of microbial communities

Supplementary Material

Vinicius S. Kavagutti[1, 2, #, ‡], Angelina Beavogui[1, #], Nicolas Wiart[3], Patrick Wincker[1], Pedro H. Oliveira[1, ‡]

[1]Génomique Métabolique, Genoscope, Institut François Jacob, Commissariat à l'Energie Atomique (CEA), CNRS, Université Evry, Université Paris-Saclay, 2 Rue Gaston Crémieux, 91057 Evry, France

[2] Current address: Department of Aquatic Sciences and Assessment, Swedish University of Agricultural Sciences (SLU), SE-75007 Uppsala, Sweden

[3] Université Paris-Saclay, CEA, Centre National de Recherche en Génomique Humaine (CNRGH), 91057 Evry, France

[#] Equal contribution

[‡] To whom correspondence should be addressed:

Vinicius S. Kavagutti (viniciuskavagutti@gmail.com)

Pedro H. Oliveira (pcoutool@genoscope.cns.fr)

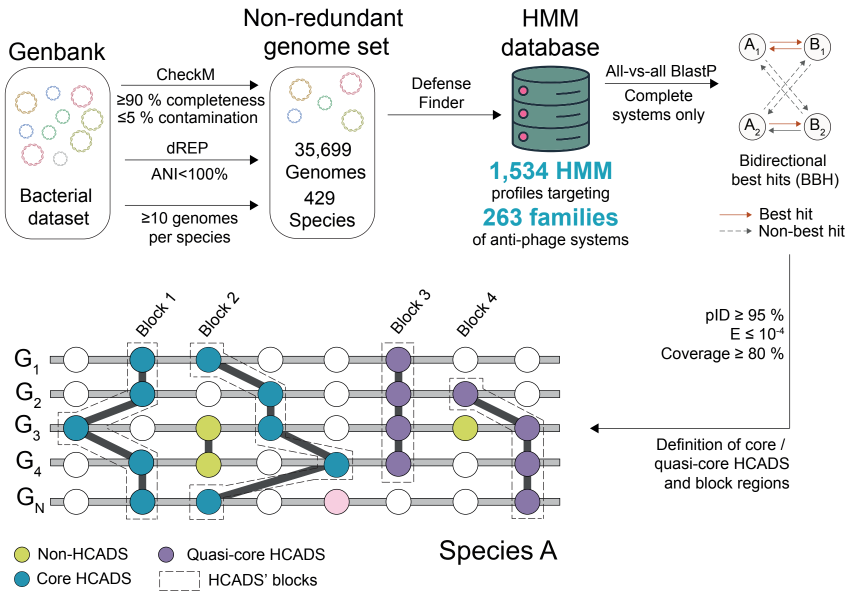

Fig. S1: HCADS analysis. HCADS identification was performed in three steps. First, a high-quality (≥90 % completeness, ≤5 % contamination) non-redundant (ANI<100 %) bacterial genome dataset (*n* = 35,699 genomes) from 429 species (those with at least 10 complete genomes) was retrieved from Genbank. Second, a collection of 1,534 HMM profiles targeting 263 families of anti-MGE defense systems from DefenseFinder, was used to query the filtered genome dataset (only complete systems were considered). Finally, a list of orthologs was identified as reciprocal best hits using BLASTP, between the defensomes of each species (pID ≥ 95 % and e-values $\leq 10^{-4}$). The core / quasi-core defensome of each species was defined as the intersection of pairwise lists of orthologs. Core and quasi-core HCADS were defined as complete defense systems, respectively present at least once in all genomes of a species, or in at least 90 % of the genomes of a species. Each set of orthologous core or quasi-core HCADS in a species was called a block.

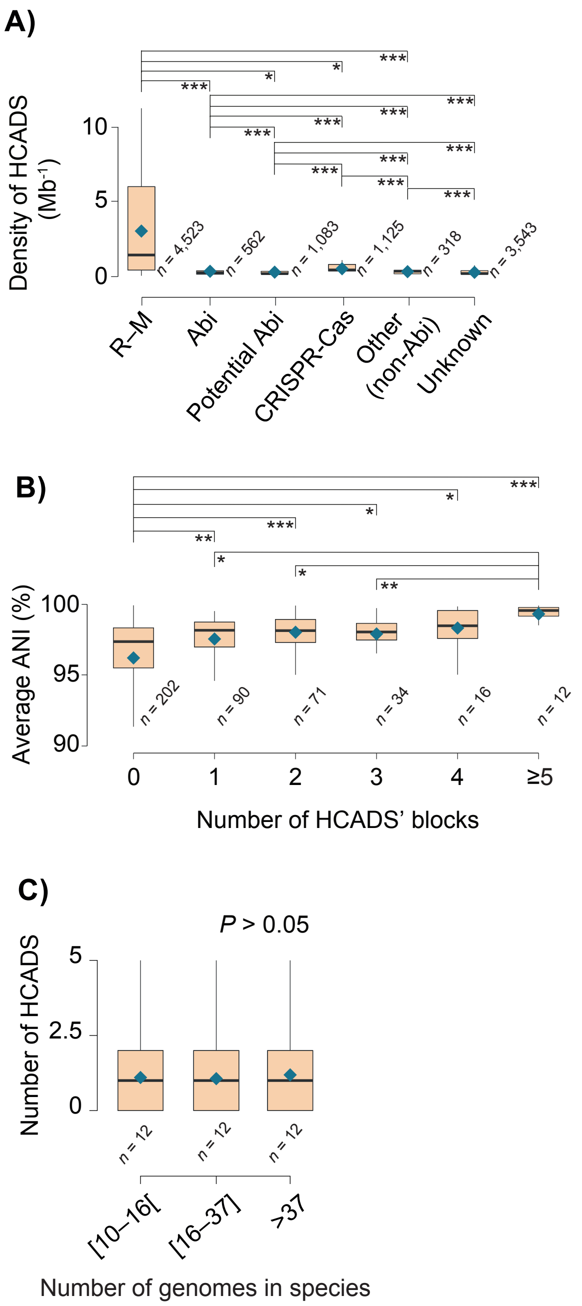

Fig. S2: Control analyses between HCADS abundance, defense mechanism, ANI, and genomes per species. (**A**) HCADS density (per genome per Mb) split per underlying defense mechanism (R–M, Abi, potential Abi, CRISPR–Cas, and other (non-Abi)). (**B**) Relation between Average Nucleotide Identity (ANI, %) and number of HCADS' blocks. (**C**) Variation in the number of HCADS with number of genomes in species. Boxplots show the 25th-75th percentiles, with the median indicated by the central black line. Whiskers extend to 1.5x the interquartile range, and individual data points were removed to improve visualization. Statistical significance was assessed using a two-sided Mann–Whitney–Wilcoxon test. *P* values are indicated as *$P < 0.05$, **$P < 10^{-2}$; ***$P < 10^{-3}$. The number of genomes (**A**, **B**) or species (**C**) analyzed are shown next to each boxplot.

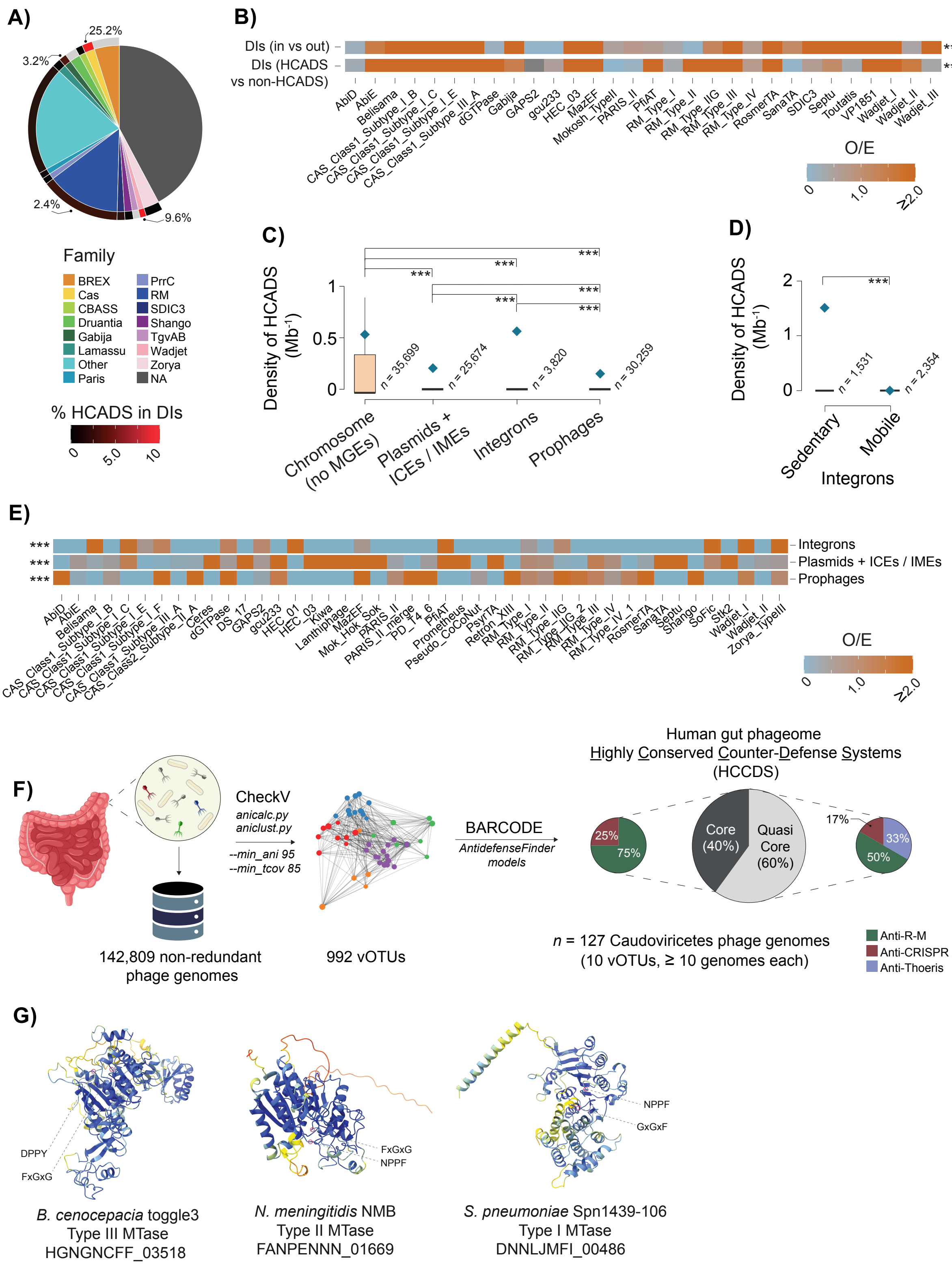

Fig. S3: HCADS, defense islands, and genetic mobility. (**A**) Pie-plot of the relative abundance (%) of defense families in DIs (inner circle). The outer layer corresponds to the abundance (%) of HCADS in DIs. (**B**) Heatmap of observed / expected (O/E) ratios of colocalization between HCADS belonging to distinct defense families and DIs. Upper heatmap represents O/E ratios between inside and outside DIs. Bottom heatmap represents O/E ratios between HCADS and non-HCADS systems inside DIs. Expected values were obtained by multiplying the total

number of HCADS in a given defense family by the fraction of HCADS from that family located within DIs. Statistical significance was assessed using a two-sided Chi-square test. Dark gray squares represent absence of colocalization. (**C**) Genomic colocalization of HCADS with plasmids + ICEs/IMEs, integrons, and prophages. Null values were included. (**D**) HCADS density (per genome per Mb) between sedentary and mobile integrons. Boxplots show the 25th-75th percentiles, with the median indicated by the central black line. Whiskers extend to 1.5x the interquartile range, and individual data points were removed to improve visualization. Statistical significance was assessed using a two-sided Mann–Whitney–Wilcoxon test. *P* values are indicated as $^{***}P < 10^{-3}$. (**E**) Heatmap of observed / expected (O / E) ratios for the colocalization of HCADS from distinct defense families with MGEs. Expected values were obtained by multiplying the total number of genes in each defense family by the fraction of genes from that family associated with each MGE. Statistical significance was assessed using a two-sided Chi-square test. (**F**) To test for the presence of Highly Conserved Counter-Defense Systems (HCCDS), we built upon the human Gut Phage Database (*n* = 142,809 non-redundant genomes). The latter were clustered (minimum 95% ANI and 80% of target coverage), yielding 992 viral Operational Taxonomic Units (vOTUs). A subsequent analysis with BARCODE using the AntiDefenseFinder option of DefenseFinder allowed to map the full phageome counter-defensome. Similarly to HCADS, HCCDS were defined as complete counter-defense systems, respectively present at least once in each genome of a vOTU, or in at least 90 % of the genomes of a vOTU. Each set of orthologous core or quasi-core HCCDS in a vOTU was called a block. (**G**) Structures of AlphaFold2-predicted HCADS' MTases belonging to three complete R–M systems in key human pathogens. Also shown are the corresponding catalytic domains (NPPY/F/W), and SAM-binding domains (FxGxxG) that can be targeted by SAM analogs for purposes of microbiome editing.
